# Supplementary material for: Transcriptomics Reveals the Putative Mycoparasitic Strategy of the Mushroom Entoloma abortivum on Species of the Mushroom Genus Armillaria
Source: mSystems. 2021 Oct 12;6(5):e00544-21. doi: 10.1128/mSystems.00544-21 (PMC8510539; doi:10.1128/mSystems.00544-21)
Supplement: TABLE S1 [file msystems.00544-21-st001.docx]

**Table S1** - Location and GenBank accession numbers of *Armillaria mellea* sequences used in the phylogenetic analysis

Country GenBank Collection/Herbarium no. Reference

Azerbaijan EF637086 B1458 1*

China KF032530 DF1 2*

China KF032532 SY1 2*

China KF032534 DJ4 2*

China KF032535 KY5 2*

China KF032536 KY4 2*

China KJ643339 HZ4 2*

China KP162319 Lijiang 3*

China: Ghizhou KT822253 HKAS86592_G_02001_5 (86)

China: Hubei KT822252 HKAS86591_G_01010_1 (86)

China: Sichuan KT822245 HKAS49004 (86)

China: Yunnan KT822242 HKAS85471 (86)

China: Yunnan KT822243 HKAS86612_K_00109_4 (86)

China: Yunnan KT822249 HKAS85599 (86)

China: Yunnan KT822251 HKAS86590_G_00020_6 (86)

China: Yunnan KT822255 HKAS86597_G_99044_2 (86)

China: Yunnan MG931746 CBSK_CFCC81073 4*

China: Yunnan MG931782 HMAS253414 4*

China: Zhejiang KT822244 HKAS86594_G_04055_9 (86)

China: Zhejiang KT822254 HKAS86593_G_04054_14 (86)

Denmark MG931762 H7000666 4*

France AF163584 B527 (87)

France AF163585 B525 (88)

France AF163586 B1245 (87)

France HQ232290 D4 5*

France JN657464 D1 5*

France JN657465 D5 5*

France MG931767 H7031840 4*

France MN660639 SREF2371 (89)

Hungary AF163581 B1212 (88)

Iran AF163583 B1205 (88)

Italy FJ716618 CRA-PAV2824 6*

Italy FJ875692 CMW 11265 (90)

Italy JF907776 8602 (91)

Italy KP960536 ER1960 7*

Italy KP960537 ER1961 7*

Italy KP960538 ER1962 7*

Italy KP960539 ER1963 7*

Italy KP960550 ER1981 7*

Italy KP960552 ER1983 7*

Italy KP960554 ER1985 7*

Italy KP960555 ER1986 7*

**Table S1** *continued*

Italy KP960556 ER1987 7*

Italy KP960557 ER1988 7*

Italy KP960558 ER1989 7*

Italy KP960561 ER1992 7*

Italy KP960562 ER1993 7*

Italy KP960563 ER1994 7*

Italy KP960564 PF1551 7*

Italy KP960565 ER1996 7*

Italy KP960566 ER1997 7*

Japan AF163594 B731 (88)

Japan KT822246 HKAS86588_83003_2 (86)

Japan MF095794 TNS:F-70421 8*

Poland AY848938 UASWS0027 9*

Poland KX756392 WA0000052261 (92)

Portugal KP960535 ER1959 7*

Scotland JF313749 TFB4184 (93)

Slovenia AJ250051 M1 (90260/1) (94)

Slovenia KF020706 17_7_9 10*

South Korea AF163591 B608 (88)

South Korea AF163592 B916 (88)

South Korea AF163593 B917 (88)

Spain EU266545 AH9328 (95)

Spain EU266546 GDA51999 (95)

Sweden KY352514 olrim1073 11*

Tibet MG931786 HMAS253585 4*

Tunisia KU863550 Oe8 12*

United Kingdom AF163578 B176 (88)

United Kingdom AF163579 B1247 (87)

United Kingdom AF163580 B1240 (87)

United Kingdom AF163582 B186 (87)

United Kingdom KX618568 MCOLLINS12 13*

United Kingdom KX618569 MCOLLINS15 13*

United Kingdom KX618581 MK7 13*

United Kingdom MH855148 CBS 106.31 (96)

Ukraine JN657466 HY3 5*

USA FJ664593 VI 49.5.1 14*

USA: California AF163595 B927 (88)

USA: California AF163596 B931 (88)

USA: California AF163597 B1217 (88)

USA: California FJ664595 PVI 85.43.1 14*

USA: California FJ664596 PVI 84-45.1 14*

USA: Georgia MG931920 TENN054336 4*

USA: Massachusetts AF163589 B496 (88)

USA: Massachusetts AF163590 B497 (88)

**Table S1** *continued*

USA: Massachusetts AY789081 PBM2470 (97)

USA: Massachusetts MF161188 FH:BHI-F169 (98)

USA: Minnesota MG931908 MIN0877204 4*

USA: New Hampshire AF163587 B282 (88)

USA: New Hampshire AF163588 B623 (88)

USA: New Hampshire AY213587 ST21 (99)

USA: New Hampshire KT822256 HKAS86611_B275 (86)

USA: South Carolina AY526602 GA.Apple clone 1 (100)

USA: South Carolina AY526603 SC.JC-3.02 clone 1 (100)

USA: South Carolina AY526604 SC.JC-3.02 clone 2 (100)

USA: South Carolina MG931929 TENN061702 4*

USA: Tennessee MF755264 JMC038 (TENN) 15*

USA: Virginia AY213584 ST5-A (99)

USA: Virginia AY213585 ST5-B (99)

USA: Wisconsin AY213586 ST20 (99)

Outgroup Taxa

*Armillaria gemina* FJ664586 II JB-38A.1 14*

*Armillaria sinapina* FJ664609 V 48.5.1 14*

*Armillaria puiggarii* KU170954 MCA 3111 (13)

*Armillaria luteobubalina* FJ664591 V M 5.2.1 14*

1*: Aghayeva DN, Harrington TC, unpublished

2*: Huang W, Zhu G, Liu C, unpublished

3*: Guo S-X, unpublished

4*: Liang J, Pecoraro L, and Cai L, unpublished

5*: Tsykun T, Prospero S, Rigling D, unpublished

6*: Scire, Motta and D’Amico, unpublished

7*: Tizzani L, Haegi A, Motta E, unpublished

8*: Kasuya,T., Maruyama,T., Fuse,K., Hosaka,K. and Minowa,K., unpublished

9*: Calmin,G., Belbahri,L. and Lefort,F., unpublished

10*: Piskur B, Hauptman T, unpublished

11*: Smith,G.R., Finlay,R., Stenlid,J., Vasaitis,R. and Menkis,A., unpublished

12*: Gharbi Y, unpublished

13*: Denman,S., Barett,G., Kirk,S.A., McDonald,J.E. and Coetzee,M.P.A., unpublished

14*: Stefani FOP, Berube JA, Hamelin RC, unpublished

15*: Matheny,P.B. and Birkebak,J.M., unpublished
